# Deciphering the ghost proteome in ovarian cancer cells by deep proteogenomic characterization

**DOI:** 10.1038/s41419-024-07046-1

**Published:** 2024-09-30

**Authors:** Diego Fernando Garcia-del Rio, Mehdi Derhourhi, Amelie Bonnefond, Sébastien Leblanc, Noé Guilloy, Xavier Roucou, Sven Eyckerman, Kris Gevaert, Michel Salzet, Tristan Cardon

**Affiliations:** 1 Univ. Lille, Inserm, CHU Lille, U1192, Protéomique Réponse Inflammatoire Spectrométrie de Masse - PRISM, F-59000 Lille, France; 2 VIB Center for Medical Biotechnology, VIB, Ghent, 9052 Belgium; 3 Department of Biomolecular Medicine, Ghent University, Ghent, 9052 Belgium; 4 Université de Lille, Inserm/CNRS UMR 1283/8199, Pasteur Institute of Lille, EGID, Lille, France University of Lille, Lille, France; 5 Department of Metabolism, Digestion and Reproduction, Imperial College London, London, UK; 6 Department of Biochemistry and Functional Genomics, Université de Sherbrooke, Sherbrooke, Québec J1E4K8 Canada

**Keywords:** Ovarian cancer, Proteome, Protein-protein interaction networks

## Abstract

Proteogenomics is becoming a powerful tool in personalized medicine by linking genomics, transcriptomics and mass spectrometry (MS)-based proteomics. Due to increasing evidence of alternative open reading frame-encoded proteins (AltProts), proteogenomics has a high potential to unravel the characteristics, variants, expression levels of the alternative proteome, in addition to already annotated proteins (RefProts). To obtain a broader view of the proteome of ovarian cancer cells compared to ovarian epithelial cells, cell-specific total RNA-sequencing profiles and customized protein databases were generated. In total, 128 RefProts and 30 AltProts were identified exclusively in SKOV-3 and PEO-4 cells. Among them, an AltProt variant of IP_715944, translated from *DHX8*, was found mutated (p.Leu44Pro). We show high variation in protein expression levels of RefProts and AltProts in different subcellular compartments. The presence of 117 RefProt and two AltProt variants was described, along with their possible implications in the different physiological/pathological characteristics. To identify the possible involvement of AltProts in cellular processes, cross-linking-MS (XL-MS) was performed in each cell line to identify AltProt-RefProt interactions. This approach revealed an interaction between POLD3 and the AltProt IP_183088, which after molecular docking, was placed between POLD3-POLD2 binding sites, highlighting its possibility of the involvement in DNA replication and repair.

## Introduction

Historically, protein sequence databases have only considered proteins to originate from the coding regions of mRNA molecules (CDS) [1, 2]. However, we now know that the sequences of many products of transcript translation are not stored in such databases [3]. Such translated transcripts include small open reading frames (smORFs) [4–6], which translate to short encoding proteins (SEPs) [7, 8] with a length of <100 amino acids. Additionally, alternative proteins (AltProt) [9–11] are translated from alternative ORFs (AltORFs) present in non-coding regions, including the 5’ and 3’UTRs, overlapping a CDS with a +1 or +2 reading, or present in non-coding RNAs (ncRNAs). In contrast to SEPs, AltProts are not limited to a maximum length of 100 amino acids. Synthesis of such proteins may result from leaky scanning and reinitiation of ribosomes as described by Marylin Kozak [12, 13]. However, such underlying mechanisms remain poorly understood and, importantly, they were not considered when the first protein databases were built, explaining the absence of quite some protein sequences in the most-often used protein sequence databases. Nevertheless, an effort has been made to make such databases more comprehensive, notably by integrating predicted protein sequences (TrEmbl) [14] which increase the size of the (theoretical) proteome. Yet, the used prediction rules are restrictive and do not consider the concept of AltProts. To tackle this, databases holding predicted sequences for AltProts such as OpenProt [9, 15] have been created. With such databases, AltProts can now be identified from bottom-up proteomic datasets. However, although such databases consider the presence of the “ghost proteome”, they do not consider mutations and neither the transcriptomic expression of samples. To overcome these limitations, OpenCustomDB [16], is a new tool that uses RNA-seq data to generate sample-specific protein sequence databases incorporating AltProts and their genetic variants. Such a proteogenomic approach coupled with AltProt research, is therefore expected to provide more comprehensive views on cellular proteomes.

AltProts are ubiquitously expressed in cells and can carry physiological functions [17]. Several AltProts have been linked to several pathways such as protein synthesis [18–20], DNA repair [8] and innate immunity [17]. AltProts have also been linked to pathologies [21, 22] such as cancers (glioblastoma, breast, ovarian and colorectal cancer) [23–26] and amyotrophic lateral sclerosis (Alt-FUS) [27]. Although their identification has been facilitated by specific enrichment and detection strategies [19, 28–30], for the overall majority of AltProts, their functions remain to be elucidated, yet targeted approaches have shed light on the function of a few AltProts [20, 29, 31–33]. Recently, we have demonstrated the effectiveness of a protein cross-linking strategy coupled to mass spectrometry (XL-MS) to annotate AltProt functions. XL-MS enabled us to identify interactions that are very close in space from 5.3 Å [34] to 30 Å [35], and by identifying cross-linked peptides between AltProts and known proteins, it completed our understanding of the function of these new proteins.

Ovarian cancer (OvCa) is considered a stealth killer due to its misdiagnosis and extended chemoresistance to treatment. In 2021, OvCa was the 8th most frequently diagnosed and source of fatal cancer in women [36]. The high mortality rate of OvCa is related to its late detection. In the initial stages of the pathology, few unspecific symptoms are evident, and diagnostic methods are not sufficiently effective [37]. The current standard treatment is based on surgery or chemotherapy. For advanced-stage tumors, debulking surgery and subsequent adjuvant chemotherapy is needed (carboplatin combined with paclitaxel is most commonly used). With this combination of treatments, up to 80% of patients will go into remission, but around 65% will relapse. Radical strategies such as oophorectomy and salpingectomy are recommended to avoid recurrence [38].

Among the metabolic pathways involved in cancer. The Kyoto Encyclopedia of Genes and Genomes (KEGG) [39] summarized different metabolic pathways. Among them, the central carbon metabolism in cancer (hsa05230) summarizes the metabolic changes that take place in cancer cells to facilitate their growth and survival [40]. This pathway involves the conversion of glucose and glutamine into intermediate molecules, which are then used to synthesize the necessary macromolecules for the replication of cancer cell biomass and genome. The Warburg effect [41], a key feature of this pathway, is characterized by the heightened utilization of glucose and glutamine by cancer cells. This phenomenon describes the extensive glucose consumption, high rates of glycolysis, and conversion of a significant portion of glucose into lactic acid even in the presence of sufficient oxygen [42]. More recently, it has been realized that the Warburg effect also encompasses an increased reliance on glutamine. Along the signaling pathways that regulate c-MYC, HIF-1, and p53, numerous other oncogenes and tumor suppressor genes are clustered [40].

For this study, we selected three cell line models to investigate differences in the reference proteome, novel isoforms and the alternative proteome. Two of these cell lines (PEO-4 and SKOV-3 cells) are derived from ascitic fluid from ovarian adenocarcinomas. Particularly, PEO-4 cells have high-grade serous histology and were collected after clinical resistance from a patient who previously received cisplatin, 5-fluorouracil and chlorambucil treatment [43]. PEO-4 cells have been xenografted into immune-deprived mice and found to be tumorigenic [44]. SKOV-3 cells are clear cell carcinoma cells and resistant to tumor necrosis factor, diphtheria toxin, cisplatin and adriamycin [45]. According to Hernandez et al. [46] and Hallas-Potts et al. [47], PEO-4 cells have a lower tumorigenicity than SKOV-3 cells when injected in nude mice. The T1074 ovarian cancer cell line was immortalized by SV40 virus and originally derived from normal human ovarian surface epithelial cells.

We hypothesized that molecular characterization of OvCa at the proteomic level might help to improve patient care and treatment. In this context, studying AltProts may shed light on mechanisms that are not yet completely understood and have an impact on OvCa pathology. This approach allowed us to identify differential expressions of RefProts, novel isoforms, AltProts and their transcripts. Additionally, the subcellular location, characteristics, and interactors of several AltProts were mapped.

## Material and methods

### Cell culture

Human PEO-4 ovarian cancer cells were cultured in Roswell Park Memorial Institute (RPMI) 1640 medium (Thermo Fisher Scientific), supplemented with 10% fetal bovine serum (Thermo Fisher Scientific), 2 mM L-glutamine (Thermo Fisher Scientific) and 100 U/mL penicillin-streptomycin (Thermo Fisher Scientific). Human SKOV-3 ovarian cancer cells were cultured in McCoy’s 5 A (modified) medium (Thermo Fisher Scientific), supplemented with 10% fetal bovine serum and 100 U/mL penicillin-streptomycin. Human immortalized ovarian epithelial cells SV-40 (T1074) were cultured in Prigrow I medium (Applied Biological Materials), supplemented with 10% fetal bovine serum and 100 U/mL penicillin-streptomycin. The three cell lines were grown in a humidified air incubator at 37 °C under an atmosphere of 5% CO_2_. The three cell lines are tested monthly for mycoplasm contamination, and the number of passages did not exceed 12. Aliquots of three million cells were harvested by trypsin-EDTA (0.05%, phenol red) (Thermo Fisher Scientific), centrifuged at 100 x g for 5 min at 20 °C and washed three times with DPBS (Thermo Fisher Scientific).

### Cell line-specific database creation

#### Total RNA sequencing (RNA-Seq)

RNA was extracted from four replicates of three million cells from each cell line employing the NucleoSpin RNA Mini kit for RNA purification (MACHEREY-NAGEL), following the vendor’s protocol. 1 µg of RNA was utilized for library preparation using RiboNaut rRNA Depletion Kit and Rapid Directional RNAseq Kit 2.0 (PerkinElmer). Nine cycles of PCR were performed during this preparation. Library sequencing was carried out using the NovaSeq6000 sequencing platform (Illumina; SP flow cell) following a 2 × 75 paired-end mode. Demultiplexing was performed using bcl2fastq v2.20.0.422. Subsequent fastq trimming utilized trimmomatic v0.39 with parameters MINLEN:35 and AVGQUAL:20. The mapping and counting steps were executed using RSEM v1.3.1 along with STAR v2.7.3a, referencing genome version hg38 and GTF from Gencode v39. Differential analysis was conducted through DESeq2 v1.24.0, employing R v3.6.3.

#### Customized protein database generation with OpenCustomDB

RNA-Seq reads were aligned to the reference genome GRCh38.p12 using STAR version 2.7.3a with default parameters except for ‘–outSAMprimaryFlag: AllBestScore,–outFilterMismatchNmax: 5, –alignSJoverhangMin 10, –alignMatesGapMax 200 000, –alignIntronMax 200 000, –alignSJstitchMismatchNmax “5-1 5 5”,–bamRemoveDuplicatesType UniqueIdenticalNotMulti’. Transcript expression was quantified in transcripts per million (tpm) with Kallisto version 0.46.0 with default parameters. Variant calling files (VCF) were generated from BAM files with FreeBayes version 1.3.1 with the setting “–min-alternate-count” set to 5. SNPs and Indels with FreeBayes quality of <20 were filtered out with an internal Python script. Variations were inserted in the corresponding transcripts with the variant annotator OpenVar. Next, the transcripts quantified by Kallisto were arranged in descending order based on their expression level (top 100,000 transcripts). Subsequently, OpenProt-annotated proteins linked to these transcripts were incorporated into the customized database until 100,000 entries (100 K DB) were reached, as described by Guilloy et al. [16]. Upon adding a protein variant to the database, the corresponding reference protein without any variation was simultaneously included to account for potential heterozygosity.

### Chemical protein cross-linking and subcellular fractionation

#### In cellulo chemical cross-linking

The cross-linking methodology performed exactly as it was described by Garcia-del Rio et al. [17, 30]. Three million cells of each cell line in triplicate were resuspended in 200 µL of DPBS. The cross-linking reaction was carried out with 2 mM (final concentration) of disuccinimidyl sulfoxide (DSSO, Thermo Fisher Scientific) at 37 °C for 1 h.

#### Protein subcellular fractionation

The subcellular fractionation methodology was perform as described in our previous work [17, 30]. In brief, the cells that underwent cross-linking were pelleted and the supernatant was removed. The Subcellular Protein Fractionation Kit for Cultured Cells (Thermo Fisher Scientific) was used to isolate five distinct protein cell compartments: cytoplasmic, membrane, nuclear, chromatin-bound and cytoskeletal proteins. Each fraction was extracted following the manufacturer’s instructions and stored at -80 °C until use.

#### Filter Aided Sample Preparation (FASP) and digestion

Each subcellular fraction was transferred to a 50 kDa molecular weight cut-off Amicon filter (Merck) and concentrated by centrifugation. Proteins were denatured, reduced, alkylated and washed in the filter. Sequential digestion was performed in each fraction. First by using trypsin/Lys-C Mix, Mass Spec Grade (Promega) at 37 °C overnight. Followed by chymotrypsin, Sequencing Grade (Promega) at room temperature for 4 h. The resulting peptides were recuperated by centrifugation, acidified with and vacuum dried. The detailed methodology can be found at our previous work [17, 30].

### Nano LC-MS/MS analysis

The resulting peptides were resuspended in 20 µL of 0.1% TFA and desalted using a ZipTip with C18 resin (Merck). Afterward, the samples were vacuum-dried and resuspended in 20 µL of a solution containing acetonitrile (ACN, Carlo Erba Reagents) and 0.1% formic acid (2:98 v/v, TCI America). Five microliters of the resulting peptide solution were analyzed on a nanoAcquity (Waters) coupled to a QExactive mass spectrometer (Thermo Fisher Scientific), as described in [24].

### Label-free quantification (LFQ) data analysis

#### Processing workflow

The raw data obtained by nanoLC-MS/MS analysis were analyzed using Proteome Discoverer V2.5 (Thermo Fisher Scientific). For each subcellular compartment, a different LFQ analysis was performed. Here, three processing steps (for each cell line’s replicates) were employed using Minor Feature Detector and three iterative Sequest HT nodes (Supplementary Fig. 1A). The detailed parameters of the Sequest HT node are described in ref. [30]. In the first Sequest HT node, the top 100,000 sequences derived from RNA-seq experiments (100 K DB) were utilized. Next, a percolator with a relaxed 0.05 FDR and strict 0.01 FDR was applied. A spectrum confidence filter was applied before moving on to the next Sequest HT node, discarding any spectra with a confidence rating worse than high. In the second Sequest HT node, the full transcript-derived database (Full DB) from OpenCustomDB was used, minus the sequences contained in the 100 K DB. The same parameters were used for a second percolator and spectrum confidence filter. Finally, in the third Sequest HT node, OpenProt was used to interrogate the sequences not found in the two previous databases (Supplementary Fig. 1B).

#### Consensus workflow

The five different subcellular fractionation MSF files were subjected to independent consensus workflows. At the feature mapper node, chromatographic alignment was performed with a maximum retention time shift of 10 min, 10 ppm mass tolerance and coarse tuning. Unique and razor peptides were used at the precursor ions quantifier node. Protein groups were considered for peptide uniqueness and shared quant results were used. Precursor abundance was based on intensity without any threshold. The total peptide amount was used for normalization mode without scaling mode. All peptides were used for normalization and protein roll-up. Modified peptides (methionine oxidation, N-terminus acetylation and cysteine carbamidomethylation) were excluded for pairwise ratios. At the PSM grouper node, the site probability threshold was set to 75. The strict and relaxed FDRs were set at 0.01 and 0.05, respectively, at the peptide validator node. Validation was based on the *q*-value, and automatic target/decoy selection was used for PSM level FDR calculation based on score. At the peptides and protein filter node, the peptide confidence was set to medium with six amino acids per peptide. Additionally, a minimum of one peptide was set. A strict (0.01) and relaxed (0.05) FDR confidence threshold were set at the protein FDR validator. The results were filtered for RefProts, AltProts and novel isoforms [9]. Briefly, a RefProt is a protein matching an NCBI RefSeq, Ensembl or UniProt protein entry. A novel isoform is a protein encoded by the same gene as a RefProt with a significant level of identity (over 80% of protein sequence identity with the RefProt over 50% of the length). An AltProt does not have any significant similarity with a RefProt.

#### Protein identification

The master protein files were uploaded as a text file to Perseus v.1.6.10.43. The abundance matrix was annotated into three categories based on the cell lines used: SKOV-3, PEO-4 and T1074. Next to count an identification, proteins needed to be identified in 70% of the replicates from at least one cell line and the groups were averaged. A numeric Venn diagram was used to identify the unique RefProts, AltProts and novel isoforms in each compartment for each cell line.

#### Statistical analysis workflow

The master protein files were uploaded as a text file to Perseus v.1.6.10.43. As a first step, log2 transformation and categorical annotation were performed on the normalized abundance values matrix, with cell lines SKOV-3, PEO-4 and T1074. To consider a valid identification, proteins needed to be identified in 70% of the replicates from each cell line. Moreover, missing values were replaced with low values of the normal distribution. An ANOVA multiple-sample test was performed using a Benjamini-Hochberg FDR *q*-value cutoff of 0.05. Non-significant values were filtered out, and a Z-score processing was applied without grouping. To ensure quality control, a principal component analysis (PCA) was conducted with a Benjamini-Hochberg FDR cutoff of 0.05. Finally, hierarchical clustering employing Pearson correlation was applied to the averaged Z-scores to identify the different protein clusters.

### Cross-linking data analysis

#### Processing workflow

The RAW data obtained by nano LC-MS/MS analysis were analyzed using Proteome Discoverer V2.5 (Thermo Fisher Scientific). The detailed parameters for the Sequest HT and XlinkX nodes are described in [24]. The triple Sequest HT nodes mentioned earlier were utilized. Instead of a percolator, a target decoy PSM validator was used after each Sequest HT node. A concatenated target decoy strategy was employed, with strict (0.01) and relaxed (0.05) FDR targets.

#### Consensus workflow

The resulting cross-linking MSF files were submitted to a consensus workflow of which the parameters are described in detail in [24].

## Results

### Differential gene expression analysis

To generate customized databases, we conducted total RNA-Seq analyses, identifying 117,636 transcripts expressed in 70% of four replicates across cell lines. Among these transcripts, 1567, 2391, and 1780 transcripts were only identified in T1074, PEO-4 and SKOV-3 cells respectively (Supplementary Fig. 2A). Total RNA-seq data analysis showed that 37,197 transcripts were differentially expressed (DESeq2, FDR < 0.05). Hierarchical clustering (Supplementary Fig. 2B and Supplementary Table 1) indicated six main transcript clusters: upregulation in PEO-4 (cluster 1, 3117) in SKOV-3 (cluster 2, 3220), or in both cell lines (cluster 3, 1138 transcripts); and downregulation in SKOV-3 (cluster 4), in PEO-4 (cluster 5), and in both cancerous cell lines (cluster 6, 12,129 transcripts). Mapping RNA-Seq reads to the human genome Hg38 identified 29,245 expressed genes across the three cell lines. Among these, 420, 407 and 540 genes were specific for T1074, SKOV-3 and PEO-4 cells, respectively (Supplementary Fig. 3A). Supplementary Fig. 3B illustrates gene annotations, where the majority were non-coding (pseudogenes and lncRNAs, 60.9%), while ~ 37% were coding genes. Hierarchical clustering based on DESeq2 results identified 17,368 genes significantly expressed (Supplementary Fig. 3C and Supplementary Table 2), with 2142 and 1949 genes upregulated in PEO-4 and SKOV-3 cells, respectively. On the other hand, 3345 and 2692 genes were downregulated in PEO-4 and SKOV-3 cells respectively, while 632 genes were upregulated and 6608 downregulated between the two cancer cell lines.

### RNA-Seq based databases

**Fig. 1:**
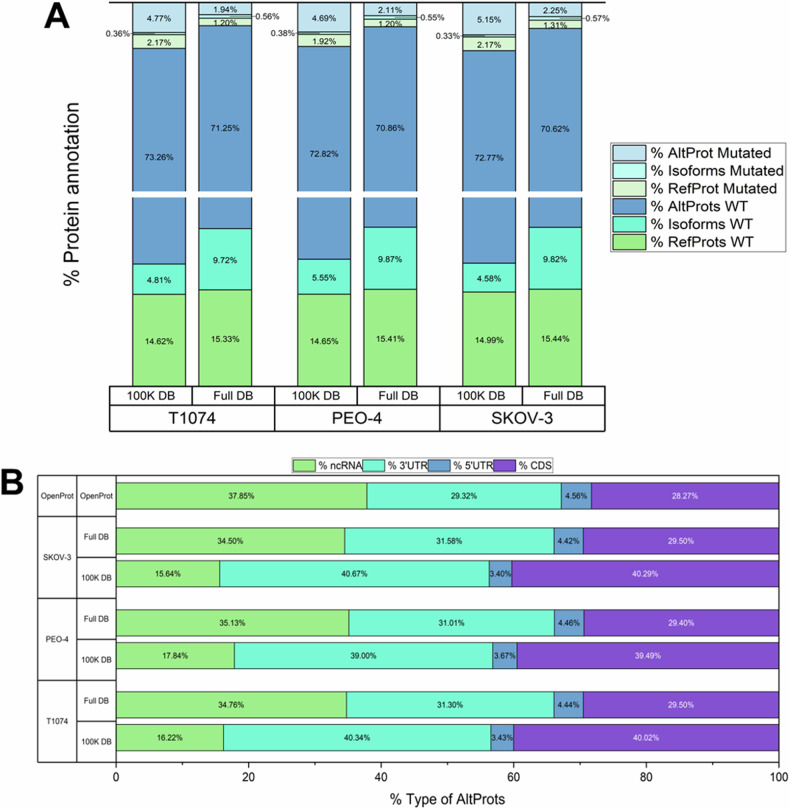
Protein prediction by OpenCustomDB. **A** WT and variant proteins predicted for each cell line. For each database, the fractions of AltProts, RefProts, novel isoforms and their variants are displayed. **B** Types of AltProts predicted by OpenCustomDB. The percentages of ncRNA, CDS frameshifts, 3’ and 5’UTR derived AltProts are displayed for each database and cell line.

Building on these findings, we utilized RNA-Seq data from T1074, PEO-4, and SKOV-3 cells to establish individualized protein databases. Figure 1A provides an overview of the protein types stored in these databases. Across all three cell lines, the custom 100 K DB comprised ~ 15% wild-type (WT) RefProts, 2% variant RefProts, 5% WT novel isoforms, less than 1% variant novel isoforms, 73% WT AltProts, and 5% variant AltProts. The OpenCustomDB workflow was employed to generate comprehensive transcript databases (Full DB) without limiting the maximum number of entries to 100,000. Specifically, T1074, PEO-4, and SKOV-3 cells contained 448,569, 443,177, and 437,568 entries, respectively. For example, in T1074 cells, 68,759 WT RefProts (15.33%), 5366 variant RefProts (1.2%), 43,609 WT novel isoforms (9.7%), 2529 variant isoforms (0.6%), 319,612 WT AltProts (71.3%) and 8694 variant AltProts (1.9%) Similar distributions were observed for PEO-4 and SKOV-3 cells (Fig. 1A). We identified the origins of predicted AltProts by mapping their transcriptomic sources using OpenProt (Fig. 1B). The main contributors to these predicted AltProts were overlap with coding sequences in shifted reading frames, as well as those found in 3’UTRs and non-coding RNAs.

Additionally, a comparison was performed between the databases across the three cell lines (see Supplementary Fig. 4). In total, 282,287 AltProts were found to overlap across the three cell lines and, 15,109, 11,026 and 8897 unique AltProts were predicted in T1074, PEO-4 and SKOV-3 cells, respectively. Among the cancerous cell lines, 8055 AltProts were found to overlap. Approximately 39,000 novel isoforms were predicted to be shared across the three cell lines, with specific novel isoforms identified in each individual cell line and both cancerous cells. Almost 60,000 RefProts overlap across all cell lines, with around 6000 being specific to each cell line.

Similar analyses were conducted on the 100 K DB (Supplementary Fig. 4), revealing 52,483 AltProts, 3116 novel isoforms and 10,346 RefProts being predicted to overlap across all three cell lines. These databases highlight specific AltProt variants present in each sample; for instance, 4321 specific AltProt variants were predicted for PEO-4 cells, 4355 for SKOV-3 and 3540 for T1074 cells. This underscores the increased presence of transcript variants in both cancerous cell lines, leading to the translation of mutated AltProts.

### Proteome analysis of subcellular compartments

**Fig. 2:**
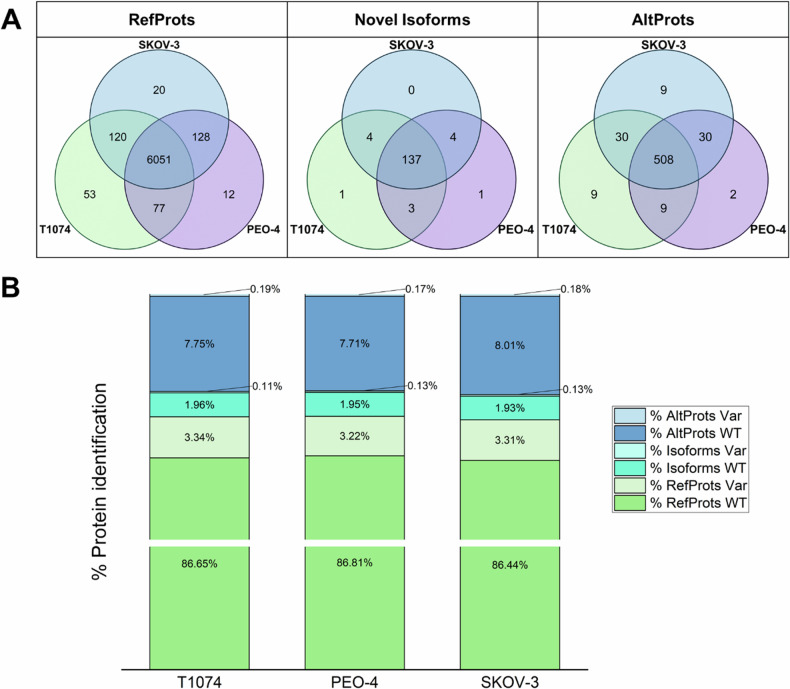
Analysis of the identified proteins. **A** Venn diagrams displaying the number of exclusive and shared proteins identified between the three cell lines. **B** Bar plot displaying the fractions of WT and variant RefProts, novel isoforms and AltProts identified in each cell line.

**Fig. 3:**
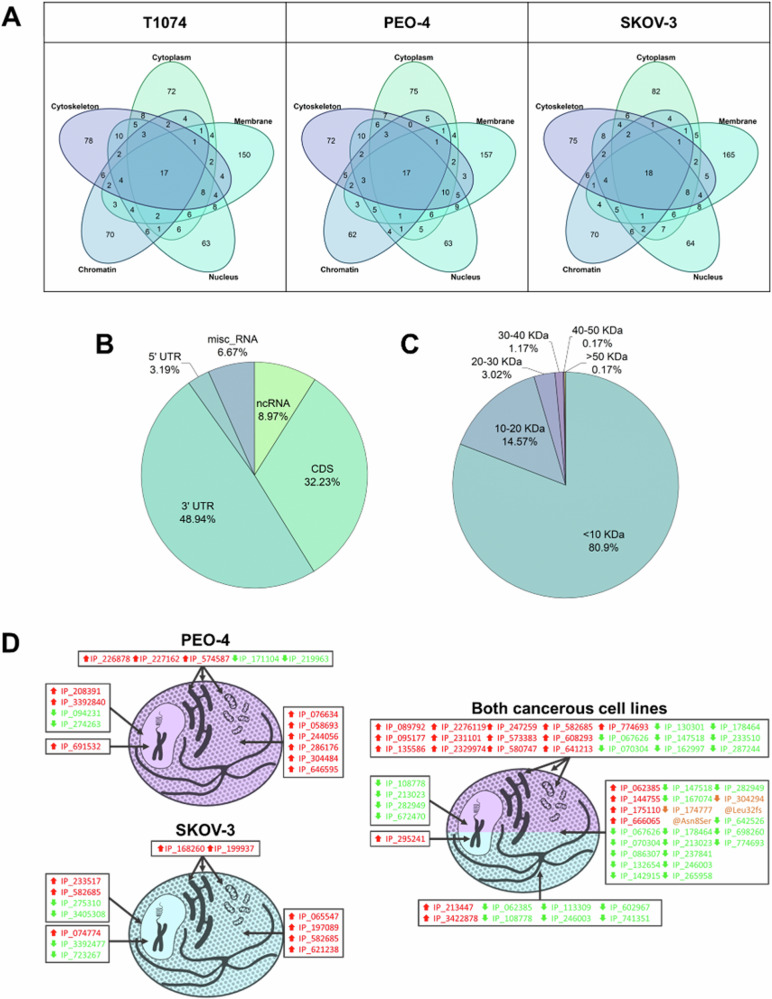
Subcellular compartment distribution, characteristics and differential expression of identified AltProts. **A** Venn diagram displaying the distribution of AltProts identified in the different subcellular fractions. **B** RNA origin and (**C**) molecular weight distribution of the identified AltProts. **D** AltProts with significantly changed levels exclusively in one of two cancerous cell lines or common in both (ANOVA, FDR < 0.05). For each cell line, the subcellular compartment, the AltProts upregulated (red) and downregulated (green) are shown.

To comprehensively assess the proteomic differences among the three cell lines, MS/MS datasets from their respective subcellular proteomes were analyzed using Proteome Discoverer V2.5. Three distinct processing workflows each consisting of three sequential Sequest HT [48] nodes were used with the databases outlined in the methods section. A protein was considered identified when it was present in at least one subcellular compartment in 70% of the replicates of at least one cell line. Figure 2A displays the distributions of all identified proteins. 6301 RefProts were identified in T1074 cells, 6268 in PEO-4 cells and 6319 in SKOV-3 cells. Among the identified RefProts, 234 (T1074 cells), 224 (PEO-4 cells) and 233 (SKOV-3 cells) were variants. For the WT RefProts, a gene ontology (GO) cellular component enrichment analysis was performed using the STRING app [49] at Cytoscape [50] (Supplementary Fig. 5). This allowed us to evaluate the fractionation. As it was explained in our previous work [17, 30], due to the principle of the kit, proteins from preceding supernatants might have been carried over into the final fraction. In addition, 137 novel isoforms were identified in T1074 cells, and 136 in PEO-4 and SKOV-3 cells. A total of 8 variants of novel isoforms were annotated in T1074 cells, and 9 in SKOV-3 and PEO-4 cells. Each cell line also displayed over 500 AltProts, with similar numbers identified in SKOV-3 cells (577), T1074 (556) and PEO-4 cells (549). Variant AltProt counts were 12 for PEO-4 cells, and 13 for both T1074 and SKOV-3 cells. Figure 2B details the distribution of wild-type and variant proteins across the samples. Subcellular fractionation was used to link cellular compartment(s) to identified AltProts (Fig. 3A). The membrane-bound fraction of all three cell lines contained the highest number of identified AltProts. In Fig. 3B, C, some general descriptions of the identified AltProts are displayed.

In addition, we identified cell line-specific RefProts, novel isoforms and AltProts. In T1074 cells, nine specific AltProts were identified, including the variant AltProt IP_290059@Asp99fs, which was found in the cytoskeletal fraction. SKOV-3 cells also had nine cell-specific AltProts, but without any variants, and PEO-4 cells had two specific wild-type AltProts identified. The characteristics of the cell line-specific AltProts are described in Supplementary Table 3. Overall, 508 AltProts were identified as shared by all three cell lines, including 11 variants.

Among the identifications, 30 AltProts were identified in both cancerous cell lines. The variant IP_715944@Leu44Pro was identified in the cytoskeletal fraction of both cell lines. The wild type AltProt IP_715944 is a 4.82 kDa protein composed of 47 amino acids and is encoded by the *DHX8* gene. The variant of this AltProt is the result of a base substitution (c.131 T > C) observed in the transcript ENST00000587574, resulting in a proline-to-leucine change at position 44.

A label-free quantitative analysis of the subcellular proteomes identified significantly altered levels of RefProts across different fractions (ANOVA, *q*-value < 0.05). Specifically, 1,022 RefProts were identified in the cytoplasmic fraction, 995 in the membrane-bound fraction, 561 in the nuclear fraction, 159 in the chromatin fraction, and 590 in the cytoskeletal fraction. Using RNA-Seq-derived databases, we also identified and quantified 88 RefProt variants with significantly different levels across the three cell lines. Among these variants, 39 were found in the cytoplasm, 39 in membrane-bound structures, 15 in the nucleus, 6 in the chromatin fraction, and 23 in the cytoskeleton. Notably, 22 of the 88 RefProt variants were present in more than one cellular fraction. Hierarchical clustering (Supplementary Fig. 6A and Supplementary Table 4) pointed to six main groups of proteins: upregulation in (1) PEO-4 cells, (2) SKOV-3 cells, and (3) in both cancerous cells; and downregulation in (4) SKOV-3 cells, (5) PEO-4 cells, and (6) both cancerous cells. Table 1 presents the number of significantly deregulated WT and RefProt variants quantified in the three cell lines.

**Table 1:** Wild-type and variant RefProts significantly varied (ANOVA, *q*-value < 0.05).

Cluster		WT RefProts	RefProt variants
Upregulated	PEO-4 cells	482	10
	SKOV-3 cells	383	6
	Both cancerous cells	666	29
Downregulated	PEO-4 cells	195	4
	SKOV-3 cells	328	16
	Both cancerous cells	1154	54

The number of WT and variant RefProts is displayed for the six main clusters identified upon LFQ proteomics.

A similar hierarchical clustering of novel isoforms identified 53 wild-type novel isoforms and three novel isoform variants with significant variation (ANOVA, *q*-value < 0.05) between the three cell lines (Supplementary Fig. 6B and Supplementary Table 5). One variant, II_587587@Asn359Asp, was upregulated in both cancerous cell lines in the cytoplasm and membrane-bound fractions. This novel isoform is expressed from the PMPCB gene. Another variant, II_702738@Ala184Thr[Leu79LeuAsn72Asn], was downregulated in SKOV-3 cells in the nuclear fraction and is encoded by the WDR18 gene, featuring a substitution at position 184 and three silent mutations. The variant II_597059@Glu65GlnAsn139AspAla57ValLys122ArgIle6ValGlu80Lys[Val118Val] was upregulated in SKOV-3 cells in the chromatin-bound fraction and is a novel isoform of HLA-H, containing seven mutations, including one silent mutation.

We used the same workflow to compare the AltProt profiles among the three cell lines. We identified 73 AltProts with significantly altered levels: 41 were upregulated in ovarian cancer cells (12 in PEO-4 only, 9 in SKOV-3 only, and 20 in both), and 36 were downregulated in both cancer cell lines, with 4 specifically downregulated in either PEO-4 or SKOV-3 (Supplementary Tables 6 and 7). Figure 3D shows the distribution of these AltProts across the five subcellular fractions. Notably, 11 AltProts were regulated in multiple compartments.

Several AltProts were downregulated in both cancer cell lines, including IP_067626, IP_070304, IP_108778, IP_147518, IP_178464, IP_213023, IP_246003, and IP_282949. Interestingly, IP_582685 (from a ncRNA transcript of the pseudogene *GDI2P1*) was upregulated in the membrane-bound fractions of both cancerous cells and also in the cytoplasmic and nuclear fractions of SKOV-3 cells. IP_062385 (translated from the 3’UTR of ENST00000457946.1 from the *ZMYM4* gene) was upregulated in the cytoplasmic fractions but downregulated in the cytoskeletal fractions of both cancerous cells. Similarly, IP_774693 (translated from an ncRNA of *TUBAP2*) was upregulated in the membrane-bound fractions but downregulated in the cytoplasmic fractions of the cancerous cells.

Two AltProt variants showed significant level differences. IP_174777, a 53-amino acid AltProt from the 3’UTR RNA of the *TMEM245* gene, had a variant IP_174777@Asn8Ser. This mutant was significantly downregulated in both cancerous cell lines compared to the epithelial ovarian cell line. The second variant, IP_304294@Leu32fs, resulted from a guanine deletion at position 93, causing a frameshift at leucine 32 and shortening the 57-amino acid protein (encoded by the *MTMR1* gene from the 3’UTR of ENST00000445323) to 44 amino acids.

### Proteome and transcriptome functional annotation

To integrate and interpret the data obtained from the differentially expressed reference proteome and transcriptome, we used the Database for Annotation, Visualization and Integrated Discovery (DAVID) [51]. The upregulated RefProts in cancerous cells showed two major cancer-related KEGG pathways [39] significantly enriched: central carbon metabolism in cancer (hsa05230; *p*-value: 1.90E-04) and chemical carcinogenesis—reactive oxygen species (hsa05208; *p*-value: 5.26E-06). The KEGG pathway proteoglycans in cancer (hsa05205; *p*-value: 0.026) was significantly enriched among the downregulated cancer RefProts.

**Fig. 4:**
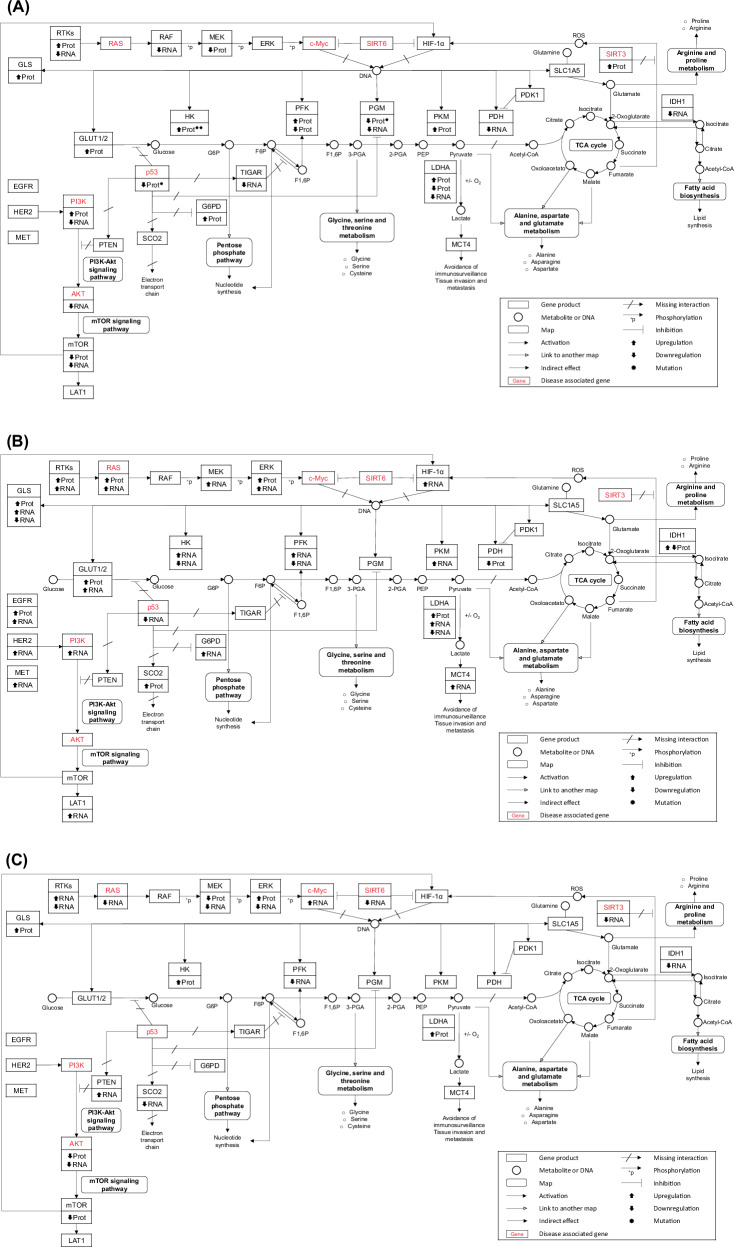
RefProts and genes significantly varied (ANOVA, FDR < 0.05) in the central carbon metabolism in the cancer pathway. **A** Central carbon metabolism in cancer, up and downregulation in both cancerous cells. **B** Central carbon metabolism in cancer, up and downregulation only in SKOV-3. **C** Central carbon metabolism in cancer, up and downregulation only in PEO-4.

The central carbon metabolism in cancer pathways (*p*-value: 7.3E-5) was found to be significantly enriched in SKOV-3. On the contrary, this pathway was not significant enriched in PEO-4 cells. Based on this difference we mapped the protein and transcript expression profiles on an adapted central carbon metabolism pathway (Fig. 4). The complete list of genes and proteins enriched for this pathway can be found in Supplementary Table 8. A significant upregulation of the NRAS protein in the RAS/RAF/MEK/ERK/c-Myc pathway was observed in SKOV-3 cells (ANOVA *q*-value: 0.017). On the other hand, its transcript levels were significantly downregulated in PEO-4 cells (ANOVA *q*-value: 0.0004). Moreover, for the other two members of the oncogene RAS family, no significant variation was found at the proteome level whereas on the transcript level, *HRAS* was downregulated in PEO-4 cells (ANOVA *q*-value: 3.7E-6) and *KRAS* upregulated in SKOV-3 cells (ANOVA *q*-value: 5.58E-5). Other differences were observed for the MEK kinases MAP2K1 and MAP2K2; for instance, MAP2K2 was significantly downregulated in both cancerous cells’ membrane-bound fraction (ANOVA *q*-value: 0.005) and downregulated in the PEO-4 cytoskeletal fraction (ANOVA *q*-value: 0.028). MAP2K1 was found downregulated in PEO-4 cells (ANOVA *q*-value: 2.29E-6) while its transcript level was found upregulated in SKOV-3 cells (ANOVA *q*-value: 1.49E-5).

In another part, SIRT6 and SIRT3 are tumor suppressor gene and considered cancer-associated genes [52–54] and downregulation of SIRT6 has been found to increase ovarian cancer cell growth [54]. The transcript levels of SIRT6, were found to be downregulated in PEO-4 cells (ANOVA *q*-value: 4.65E-6). Protein levels of SIRT3, were upregulated in both cancerous cells (ANOVA *q*-value: 0.005), while its transcript levels were found downregulated in PEO-4 cells (ANOVA *q*-value: 0.0001). The expression of the oncogenic PI3K family was also found significantly regulated among the three cell lines. PIK3R1 was upregulated in both cancerous cells’ cytoplasmic fraction (ANOVA *q*-value: 0.037), while its transcript was only upregulated in SKOV-3 cells (ANOVA *q*-value: 2.31E-5). Additionally, the transcripts of *PIK3CB* (ANOVA *q*-value: 0.0001) and *PIK3R2* (ANOVA *q*-value: 0.005) were also only upregulated in these cells. On the contrary, the *PIK3CA* (ANOVA *q*-value: 0.001) and *PIK3CD* (ANOVA *q*-value: 0.0001) transcripts were found downregulated in both cancerous cells.

AKT1 protein (ANOVA *q*-value: 0.0002) and transcript levels (ANOVA *q*-value: 2E-5) were downregulated in PEO-4 cells. For AKT2 and AKT3, no significant variation in protein expression was found, while their transcript levels were significantly downregulated in both cancerous cells (ANOVA *q*-value: 0.02 and 3.6E-6).

With our proteogenomic workflow, we could identify a variant form of p53 (ENSP00000269305.8: p.Pro72Arg), an amino acid substitution that stems from the c.215 C > G variant in *TP53*. This p53 mutant was significantly downregulated in both cancerous cells’ cytoplasmic (ANOVA *q*-value: 0.0036) and cytoskeletal (ANOVA *q*-value: 0.0096) fractions, while its transcript levels were only significantly downregulated in SKOV-3 cells (ANOVA *p*-value: 1.17E-10). Three other RefProt variants were identified in this pathway. ENSP00000359991.5: p.Thr238Met, a mutant of PGAM1 was downregulated in both cancerous cells (ANOVA *q*-value: 0.0013), while two mutants of HKDC1 were upregulated in both cancerous cells; ENSP00000346643.5: p.Thr124Ile, p.Asn917Lys, p.Arg827Trp, p.Trp721Arg, [p.Phe601Phe] (ANOVA *q*-value: 0.008) and ENSP00000346643.5: p.Thr124Ile, p.Asn917Lys, p.Trp721Arg, [p.Phe601Phe] (ANOVA *q*-value: 0.023).

### Cross-linking network analysis

The computational analysis of the cross-linked samples was carried out as described in [30], which allowed us to generate a protein interaction map in Cytoscape [50] (Supplementary Fig. 7). A total of 90 cross-links were identified (Supplementary Table 9), among them 20 intra-cross-links were identified. In this protein network (Supplementary Fig. 7), 28 protein-protein interactions (PPIs) were found in PEO-4 cells (marked in purple), 27 in SKOV-3 cells (marked in blue) and 35 in T1074 cells (marked in green). From these pairs, 12 cross-link interactions were identified in at least two cell lines. Among all the cross-linked pairs, 20 involved AltProts, four cross-links were AltProt-AltProt interactions, and 13 AltProt-RefProt cross-links were identified. The latter were considered most important for our study as they provide hints to an AltProt’s physiological or pathological involvement.

**Fig. 5:**
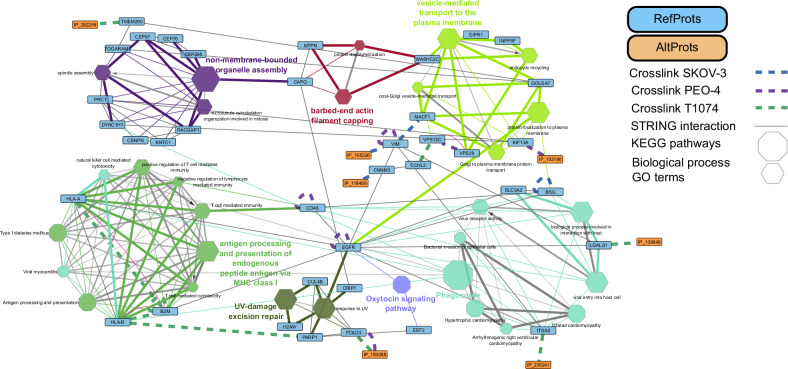
GO molecular function enrichment network generated with ClueGO in Cytoscape. GO enrichment was generated from the accession numbers of Supplementary Fig. 8. AltProts are marked in orange and RefProts in blue. Enriched GO terms are displayed as hexagons. KEGG pathways are displayed as octagons and cross-links are marked in blue (SKOV-3 cells), purple (PEO-4 cells) and green (T1074 cells) dashed lines.

To attribute functions to an AltProt from this set of PPIs, we retrieved the known interactions from the STRING [49, 55], BioGrid [56] and IntAct [57] databases and included the identified cross-linked interactions (Supplementary Fig. 8). Additionally, for the RefProts that did not present a referenced STRING interaction within the cross-linked network, the addition of three STRING interactors has been performed to expand the network. Using this network, a molecular function GO term and KEGG pathway enrichment analysis was performed with the ClueGO App [58]. The interactions between AltProts and RefProts were displayed along with the enriched GO terms (Fig. 5). Four direct AltProt-RefProt-GO-term interactions were detected. The AltProt IP_192190 was cross-linked to KIF13A in PEO-4 cells and linked to the transport GO terms (GO:0098876, GO:0043001 and GO:0072659). The AltProt IP_136846 was identified as cross-linked to LGALS1 in T1074 cells, which is linked to the GO terms viral entry into host cell (GO:0046718) and biological process involved in interaction with host (GO:0051701). Similarly, IP_235241 cross-linked to ITGA5 in T1074 cells, was linked to the phagosome KEGG pathway (KEGG:04145) and the GO terms involving viral processes (GO:0001618, GO:0051701 and GO:0046718). Finally, IP_183088 was cross-linked to POLD3 in T1074 and PEO-4 cells. POLD3 is part of the DNA polymerase involved in the replication and reparation of DNA and linked to the UV-damage excision repair (GO:0070914) and response to UV (GO:0009411) GO terms.

Three AltProt-GO term/KEGG pathways indirect links were identified. IP_292259 cross-linked to TMEM260 in T1074 cells, and TMEM260 possesses a STRING interaction with TOGARAM, which is linked to the non-membrane-bounded organelle assembly GO terms (GO:0140694, GO:0051225 and GO:1902850). Additionally, TMEM260 interacts with GOLGA7, which is linked to GO terms related to the vesicle-mediated transport to the plasma membrane. In addition, two AltProts were also identified to be related to these GO terms: IP_105326 and IP_118499. The former was cross-linked to VIM in SKOV-3 cells, and VIM was cross-linked to MACF1, which is linked to vesicle-mediated transport GO terms. IP_118499 was found cross-linked to CNNM3 in SKOV-3 cells, which processes a STRING interaction with CCNL2, which was cross-linked to VPS13C, which is linked to vesicle-mediated transport GO terms.

To confirm the probability of the observed interactions, we analyzed 3D models of RefProt-AltProts using unguided interaction docking between the two partners (as described in [30]). The structures of the AltProts were predicted using I-Tasser [59], while those of the interactors were predicted using ClusPro [60]. The RefProt, for which the structure was predicted by AlphaFold [61], was used as a receptor of the AltProt, which was smaller in structure. By measuring the distance of the predicted interactions, we confirmed the observed interactions from XL-MS with a mean of 23.467 Å (Supplementary Fig. 9), which is consistent with the distances described in the literature for DSSO, ranging from 5.3 [34] to 30 Å [35].

## Discussion

Proteogenomics establishes a direct connection between the genome blueprint and the constructed proteome. We utilized this approach to explore the potential implications of AltProts in ovarian cancer. We selected the PEO-4 cell line possessing high-grade serous histology, the SKOV-3 clear cell carcinoma cell line, and the T1074 ovarian epithelial cell line, originally derived from normal human ovarian surface epithelial cells, serving as a non-tumorous control.

Employing DESeq2 from RNA-seq data enabled us to identify clusters of regulated genes in the cancer cell models, with each cell line showing about 500 uniquely expressed genes. Among the 540 genes uniquely expressed in PEO-4 cells, the proto-oncogenes SSX1, SSX2 and SSX2B were found, along with 24 genes related to cancer according to the Gene-Disease Associations Dataset (GAD) [62]. SKOV-3 cells had 406 unique expressed genes, with 23 related to cancer by GAD. While transcriptomic analysis provided cell specificity information, the strength of this approach lies in the custom creation of cell-specific databases using OpenCustomDB. These databases contain a larger number of AltProt variants due to the high number of predicted AltProts. The ratio of variant RefProts to WT RefProts was greater than the ratio of variant AltProts to WT AltProts, which can be attributed to differences in sequence length. Longer genomic sequences have higher mutation rates and replication errors. Additionally, predicted AltProts mostly originate from ncRNAs, with mRNA CDS frame shifts and 3’UTRs also contributed significantly to the top 100,000 most abundant transcripts.

The proteogenomic approach of constructing a custom database, combined with reading frame prediction for AltProt generation, presents analytical challenges. However, our iterative triple SEQUEST HT processing workflow using the 100,000-abundance cut-off database in the first node overcomes the FDR limitations of a 400,000-sequences database (full database) search, which may increase the number of false positives and false negative identifications [16]. To not lose possible identifications, such iterative workflows provide a stepwise increase in possible protein identifications by expanding the search space, until the last step with OpenProtDB, where proteins translated from ncRNAs not detected by RNA-Seq can be recovered. Finally, using Percolator, we removed false positive identifications by this semi-supervised machine learning algorithm [63]. Percolator effectively estimates the statistical significance of peptide-spectrum matches and assigns confidence scores to identified peptides in a fast and accurate way. It enhances the rate of confident peptide identifications from a collection of tandem mass spectra [64].

Subcellular fractionation is an approach to decrease sample complexity and maximize resolution in LC-MS/MS analysis. In our previous works [17, 30], it was proven beneficial for XL-MS workflows and provided better coverage of the proteome compared to analyzing whole cell lysates [65]. This enhanced the detection of low-abundant proteins (AltProts and cross-linked proteins). Furthermore, it helps to determine the subcellular localization of AltProts and monitors changes under different cellular conditions [66]. For instance, IP_062385 was found upregulated in cancerous cells at the cytoplasm, while downregulated in their cytoskeleton fractions. This may reflect a functional change linked to cancer, yet targeted studies will be necessary to prove such links between tumor development and AltProts re-localizing over different cellular compartments. However, it is important to note that subcellular fractionation based on the use of different detergents can lead to potential cross-contamination and inaccuracies in downstream data interpretation.

**Fig. 6:**
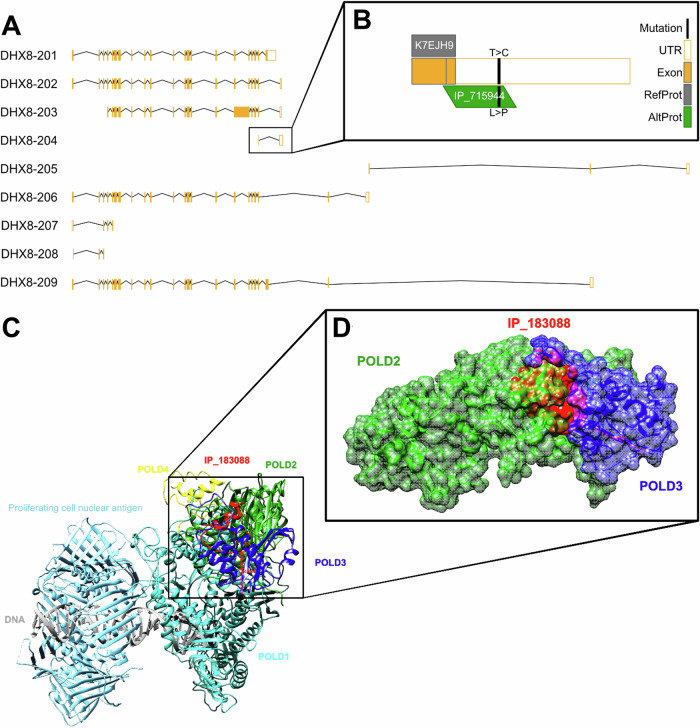
Overview of the origin of AltProts, illustrating the impact of known silent mutations on RefProts and their corresponding effects on AltProts, along with 3D modeling of AltProt-RefProt interactions as observed through XLink-MS. **A** Synthesis of AltProts from the DHX8 gene, displaying the list of transcripts referenced in Ensembl. **B** Zoom on DHX8-204 described to translate to “K7EJH9”, a predicted protein from TrEMBL without the 5’UTR part or methionine as the first amino acid, when IP_715944 is described from the overlap between the CDS and the 3’UTR. As a result, the mutation is only observable by the proteogenomic construction, as it would be considered a silent mutation due to its position in the UTR part of K7EJH9. **C** IP_183088 (AltMAPK8) predicted models docked to the human polymerase delta holoenzyme complex. The interaction of IP_183088 and the full POLD complex is shown. The distance between the two lysines involved in the cross-link is 24.59 Å. **D** Zoom of the interaction of IP_183088 and POLD3. The surface representation shows the possible placement of IP_183088 at the interaction site of POLD3 and POLD2.

Subcellular fractionation led to the identification of ~ 6000 common RefProts among the three cell lines. Over 3% of all identified proteins in each cell line were RefProt variants (Fig. 2B). However, these ~180 RefProt variants require deeper characterization to understand their (pathological) role. Cell line-specific AltProts were also identified, AltProts in SKOV-3 and PEO-4 cells are of interest as potential new protein markers for OvCa. Among them, IP_715944@Leu44Pro (Fig. 6A, B) caught our attention as it is a variant AltProt not predicted in the T1074 RNA-Seq database. Six additional AltProts from this group were also not predicted, which highlights the importance of a cell-specific analysis to identify new biomarkers.

AltProts were found to be upregulated in all compartments except the cytoskeleton of PEO-4 and SKOV-3 cells, while downregulation of AltProts was only observed in the membrane-bound and nuclear fractions in PEO-4 cells, and in the nuclear and chromatin fractions in SKOV-3 cells. When comparing both cancerous cell lines to T1074 cells, significant downregulation of AltProts was observed in all five compartments. AltProts upregulated in both cancerous cells were present in all compartments except the nucleus. These findings provide some insights into the specific expression of AltProts in high grade serous and non-serous OvCa. Functional domains were predicted for 23 out of 73 AltProts, which can help us understand their potential roles in interactions. Future targeted interactomic approaches such as Virotrap [67], BioID [68] and proximity ligation assays [69] could be used to identify the interaction partners of these AltProts, which may shed light on their involvement in the pathogenic development of OvCa or drug resistance.

NRAS, a member of the RAS oncogene family, is involved in cell signaling, regulation of cell growth, differentiation and angiogenesis. In ovarian clear cell carcinoma, no NRAS mutations were found in transcriptome data [70]. However overexpression in proteomic was shown, this have been described to increase tumor aggressiveness in mice [71]. KRAS, another member of the RAS oncogene family, was found to be upregulated in SKOV-3 cells and in metastatic lesions in endometrial cancer [72], which is associated with adverse prognosis [73]. Downregulation of HRAS has been linked to lower aggressiveness and reduced cell proliferation in certain types of cancer [46, 74, 75]. Another branch of the pathway also shows MEK (mitogen-activated extracellular signal-regulated kinase) which is a kinase cascade pathway that plays a central role in carcinogenesis and the maintenance of several cancers. We found downregulation of MAP2K1 and MAP2K2 in both cancerous cell lines, as also evident from data in The Human Protein Atlas [76]. In parallel, related to cancer metabolism, we observed *SIRT6* downregulation and *c-Myc* upregulation in PEO-4 cells. Lower levels of *SIRT6* are associated with poorer prognosis and increased tumor aggressiveness [53, 54]. *SIRT6* also regulates ribosome metabolism by repressing *c-Myc* activity. As a result, higher levels of *c-Myc*, resulting from the downregulation of *SIRT6*, promote energy production and biomolecule synthesis for rapid cell proliferation. On the other hand, *SIRT3* is described as a tumor suppressor gene in OvCa [77] and its expression increases in detached cells and tumor cells from malignant ascites, indicating its pro-metastatic role in OvCa [52]. Our proteomic data show upregulation of SIRT3 in both cancerous cells, while *SIRT3* transcripts are downregulated in PEO-4 cells. Discordance between mRNA and protein levels has been observed in various studies [78–81], attributed to post-transcriptional regulation, transcript isoform switching and DNA variants [79, 82]. We found that PIK3R1 (p85α) was upregulated in the tumoral cells, which also corresponds to the identified overexpression of PIK3R1 in an OvCa cohort of 98 patients [83]. However, contrary to literature findings [84], transcript levels of PIK3CD were downregulated in both cancerous cell lines. Stronach et al. [85] and Liu et al. [86] have studied the role of the AKT kinase signaling pathway in OvCa cell proliferation, cell cycle regulation and anti-apoptosis. They discovered that SKOV-3 cells rely on AKT1 for cisplatin resistance, while PEO-4 cells depend on AKT3. In line with this study, in our dataset, both protein and transcript levels of AKT1 were found to be overexpressed in SKOV-3 cells.

Among the significantly deregulated RefProts identified in our study, P53 rs1042522 was found downregulated in both cancer cell lines. The corresponding Pro72Arg substitution in the canonical P53 sequence (UniProtKB: P04637-1) occurs in a proline-rich, intrinsically disordered region (residues 64–92) [87]. This region is described as rigid [88] and a substitution of one of the prolines in this region might decrease its stiffness. Moreover, position 72 is part of the binding site of P53 with the oncogenic protein MDM2 [89]. Even though there is evidence suggesting that there may be an association between this mutation and OvCa risk, a meta-analysis by Schildkraut et al. could not confirm an association with OvCa [90]. Additionally, using our proteogenomic approach we were able to confirm the observations of Yaginuma et al. [91] describing SKOV-3 as a null-WT-P53 cell line.

XL-MS reveals clues about AltProt functions based on AltProt-RefProt PPIs, IP_183088, a 38-amino acid AltProt, is encoded by *MAPK8* and was found to interact with POLD3 in T1074 and PEO-4 cell lines. Figure 6C displays the model of the human polymerase delta holoenzyme complex (PDB: 6s1m). Herein, the four subunits of the complex are shown (POLD1 turquoise, POLD2 green, POLD3 blue and POLD4 yellow), additionally, the proliferating cell nuclear antigen is displayed in light blue and the AltProt IP_183088 in red, together with its cross-links. Figure 6D zooms in on the cross-linked region of POLD3-IP_183088, revealing that this interaction occurs in the region where POLD2 and POLD3 interact. Our transcriptomic data point to POLD3 downregulation in both cancerous cells. This correlates with the findings of Willes et al. who described that POLD3 downregulation is correlated with a poor cancer outcome [92] and those of Weberpals et al. who showed that POLD3 is overexpressed in patients with high-grade serous ovarian carcinoma and with good response to carboplatin/paclitaxel [93]. On the other hand, the inhibition of the interaction between POLD3 and POLD2 driven by IP_183088 can reflect two effects. (i) An increase of the mutagenesis in the cells upon reduced activity of the POLD complex and, therefore, errors in DNA replication are more likely to occur and go unrepaired, which can be expected in PEO-4 cells. (ii) A regulatory system of the POLD complex, where the POLD3-IP183088 interaction in T1074 cells could lead to cell apoptosis; Murga et al. [94] showed that POLD3 stabilizes the POLD complex and in its absence, the cell is driven to apoptosis. The difficulty of detecting interactions by XL-MS means that we cannot claim that the observed interactions are cell-type specific, but they do provide information about potential protein functions for unreferenced proteins.

To conclude, one main advantage of the databases generated by OpenCustomDB is the possibility of predicting and identifying cell-specific proteins in cell lines and, in the future, in patient samples, resulting in a big step forward toward personalized medicine. Subcellular fractionation allowed us to study differences in the reference, alternative and novel isoforms proteome of OvCa cell lines compared to a non-tumoral ovarian epithelial cell. Additionally, it allowed us to identify RefProts variants and understudied AltProts and their variants. The versatility of these databases allowed us to identify AltProt-RefProts PPIs and gave some clue about the function of AltProts, which however needs to be validated. In summary, our large-scale characterization study revealed other research targets and demonstrated the complexity of the cell proteome and its largely unmapped ghost proteome.

## Supplementary information

Supplemental Figures

Supplemental Tables

## Data Availability

“The mass spectrometry proteomics data have been deposited to the ProteomeXchange Consortium via the PRIDE [95] partner repository with the dataset identifier PXD045689”. RNAseq analysis data have been deposited to BioProject (SRA) with dataset identifier: PRJNA1041444 and GEO dataset identifier: GSE248039.
