# Supplementary material for: Deciphering the ghost proteome in ovarian cancer cells by deep proteogenomic characterization
Source: Cell Death Dis. 2024 Sep 30;15(9):712. doi: 10.1038/s41419-024-07046-1 (PMC11442847; doi:10.1038/s41419-024-07046-1)

Supplemental Figure 1. LFQ analysis workflow. (A) Illustration of the Proteome Discoverer analysis steps used. Each child processing step corresponds to the interrogation using the cell-specific database. (B) Workflow nodes present in each processing child step.

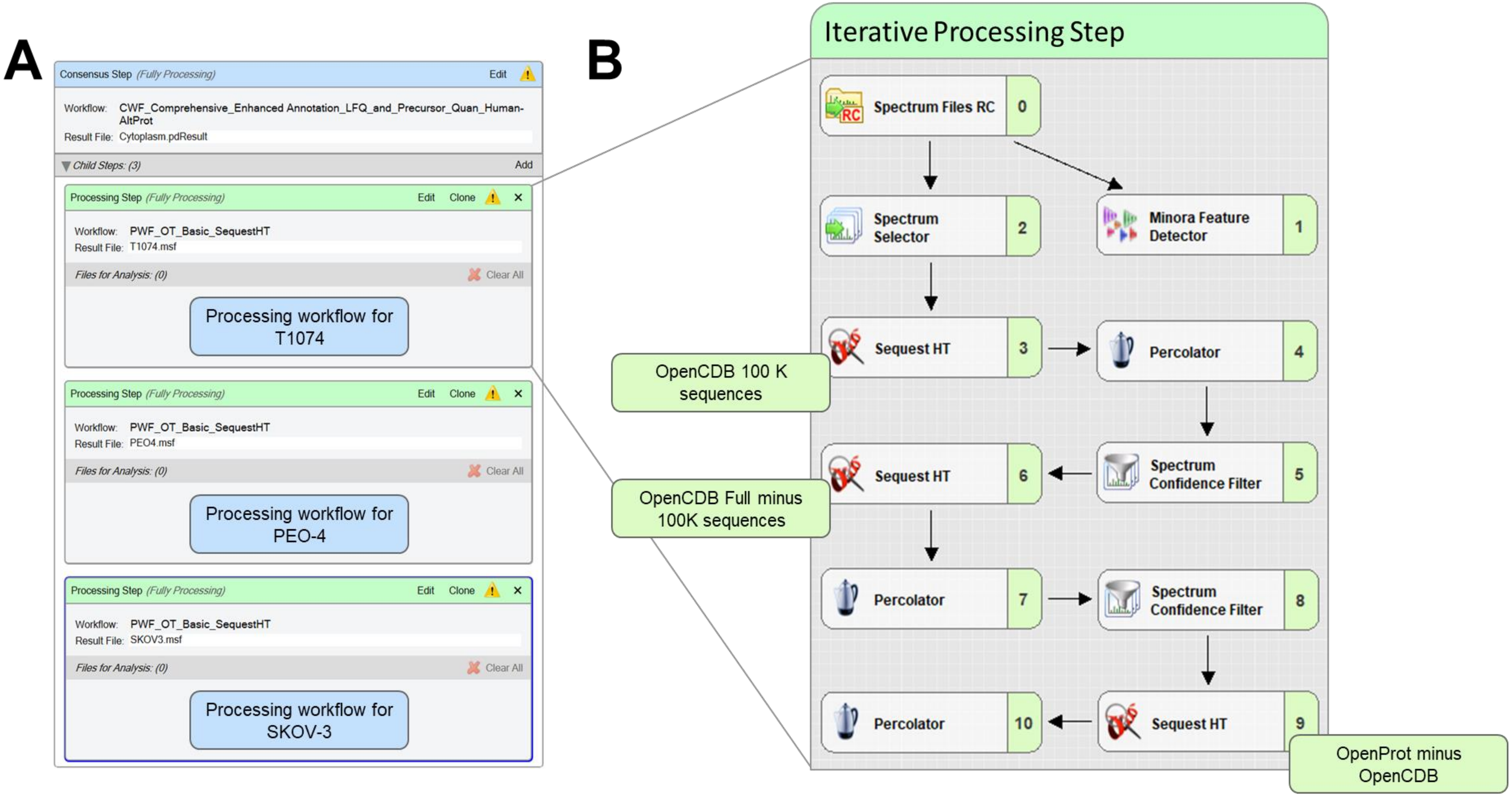

Supplemental Figure 2. DESeq2 transcripts analysis. (A) Venn diagram displaying the number of exclusive and shared transcripts between the three cell lines. (B) Hierarchical clustering heatmap showing the different transcript clusters that can be observed among the three cell lines. Z-score range from -1.3509 (green) to 1.3523 (red).

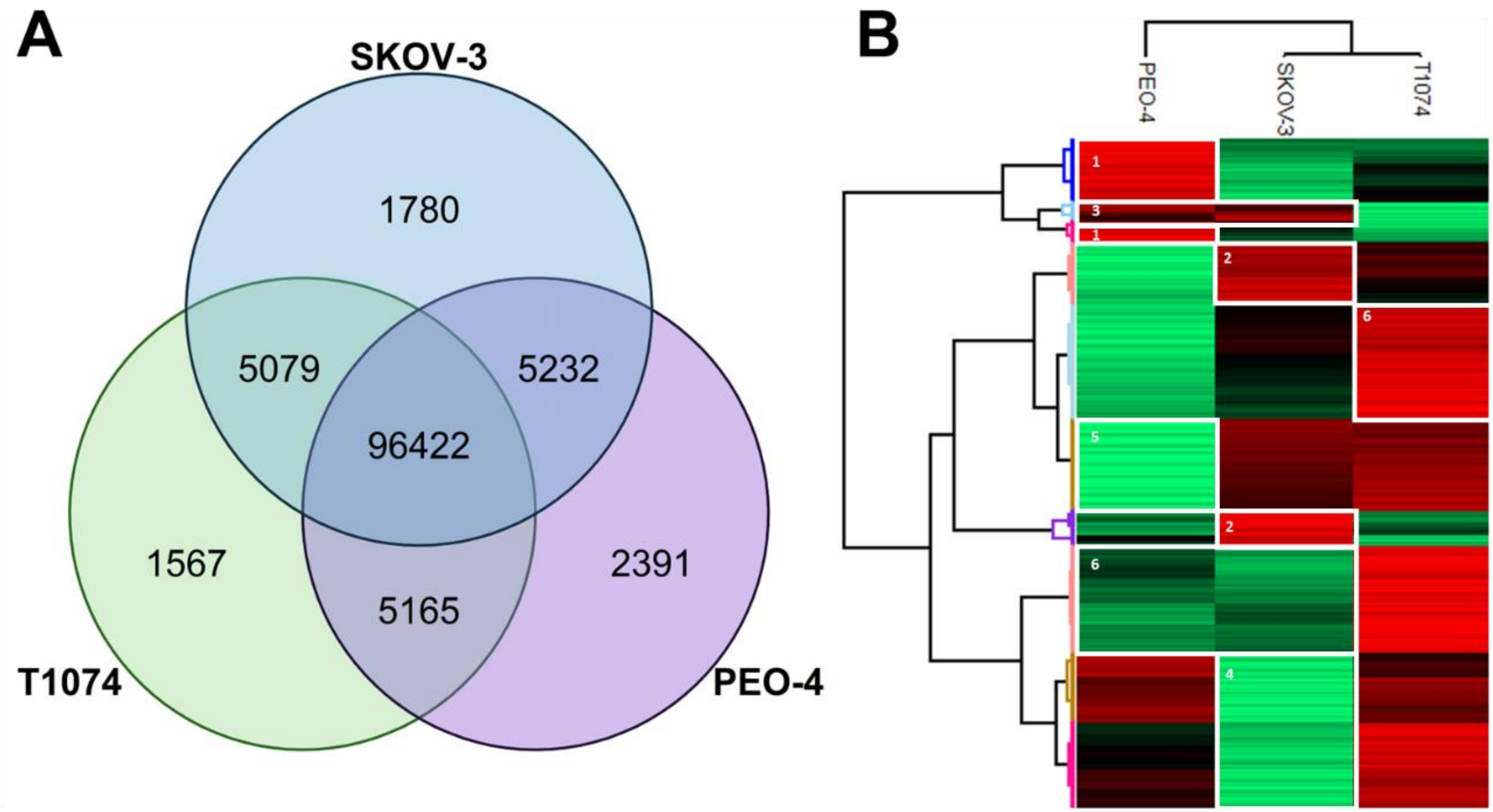

Supplemental Figure 3. DESeq2 gene analysis. (A) Venn diagram displaying the number of exclusive and shared genes between the three cell lines. (B) Pie chart displaying the ratios of the different types of RNAs sequenced. (C) Hierarchical clustering heatmap showing the different gene clusters that can be observed among the three cell lines. Z-score range from -1.351 (green) to 1.3496 (red).

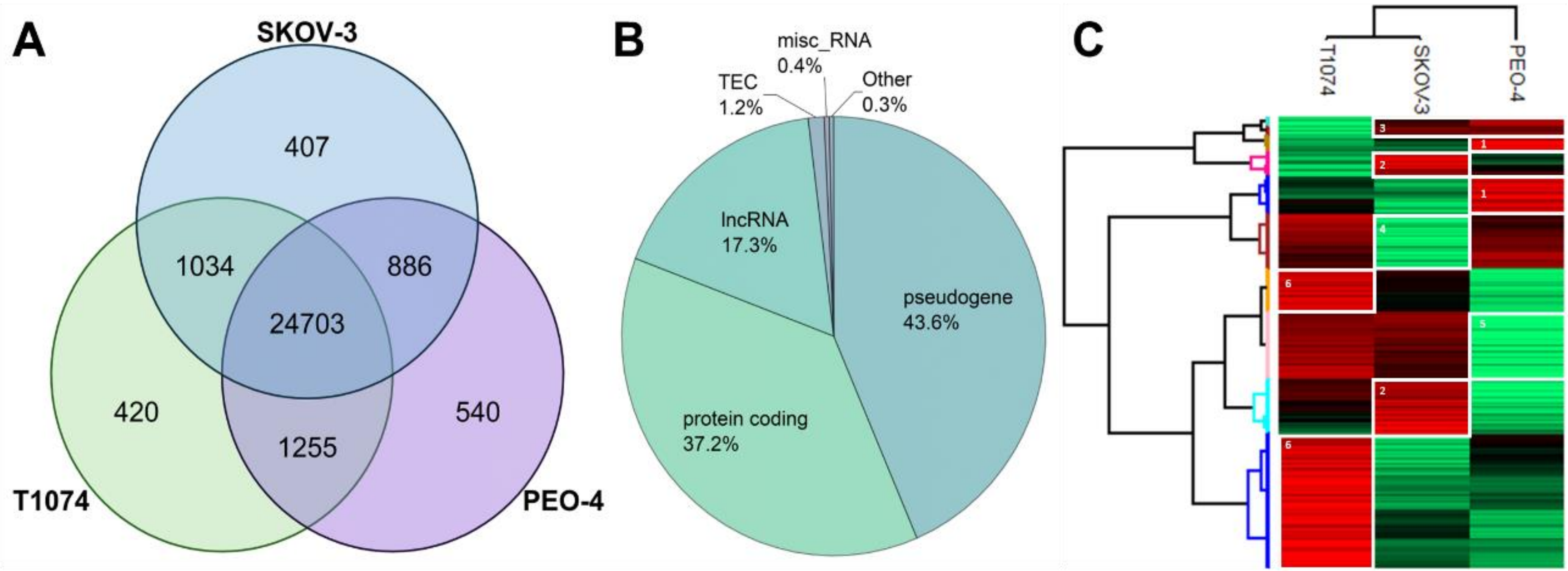

Supplemental Figure 4. Venn diagrams describing the RNA-seq derived databases. The number of specific and common proteins are shown for each cell line.

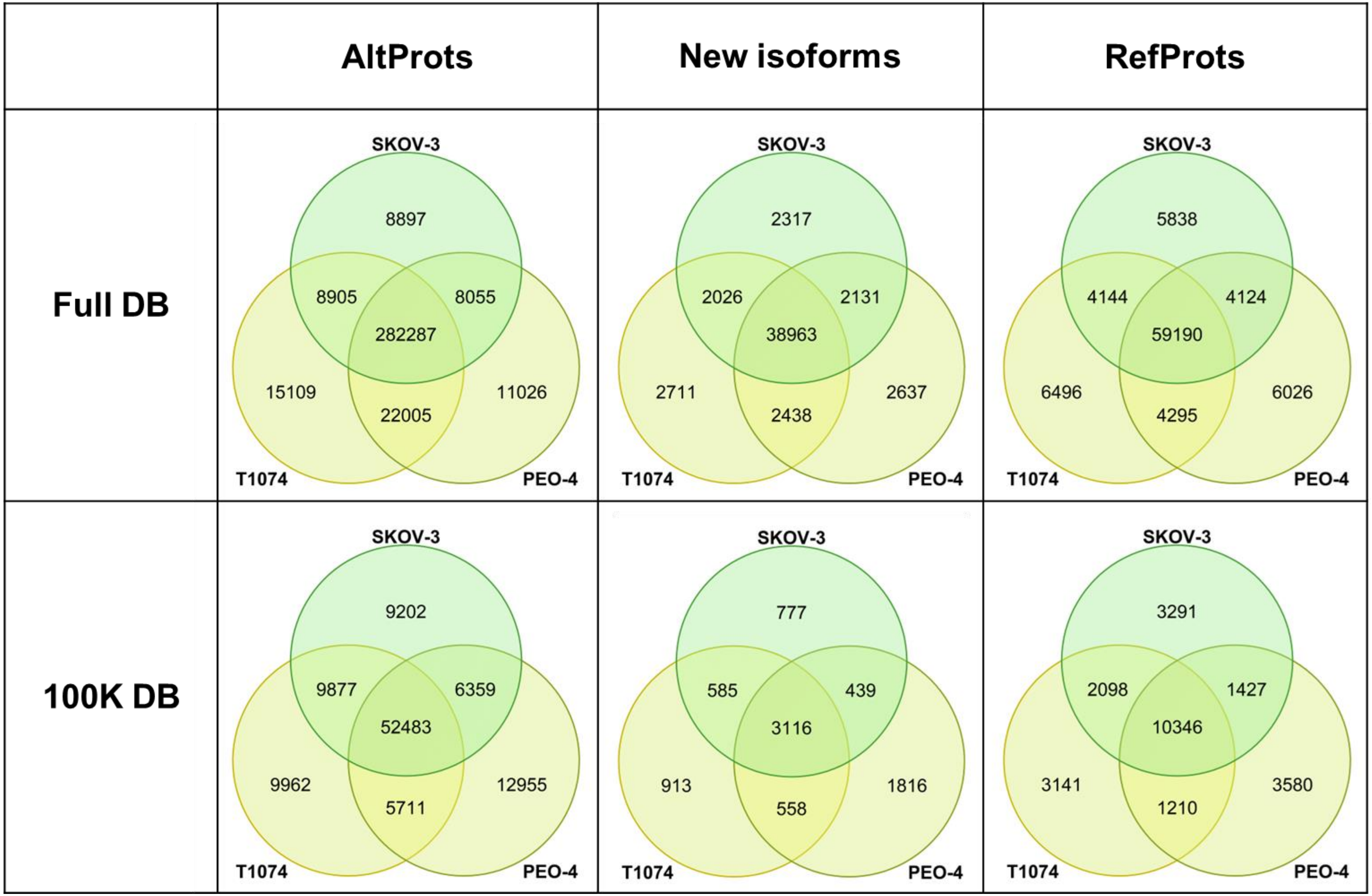

Supplemental Figure 5. Bar chart showing the number of WT-RefProts identified (green), the number of RefProts indexed in STRING (blue), and the number of RefProts that contain the GO term of the localization corresponding to the fraction where it was found (purple).

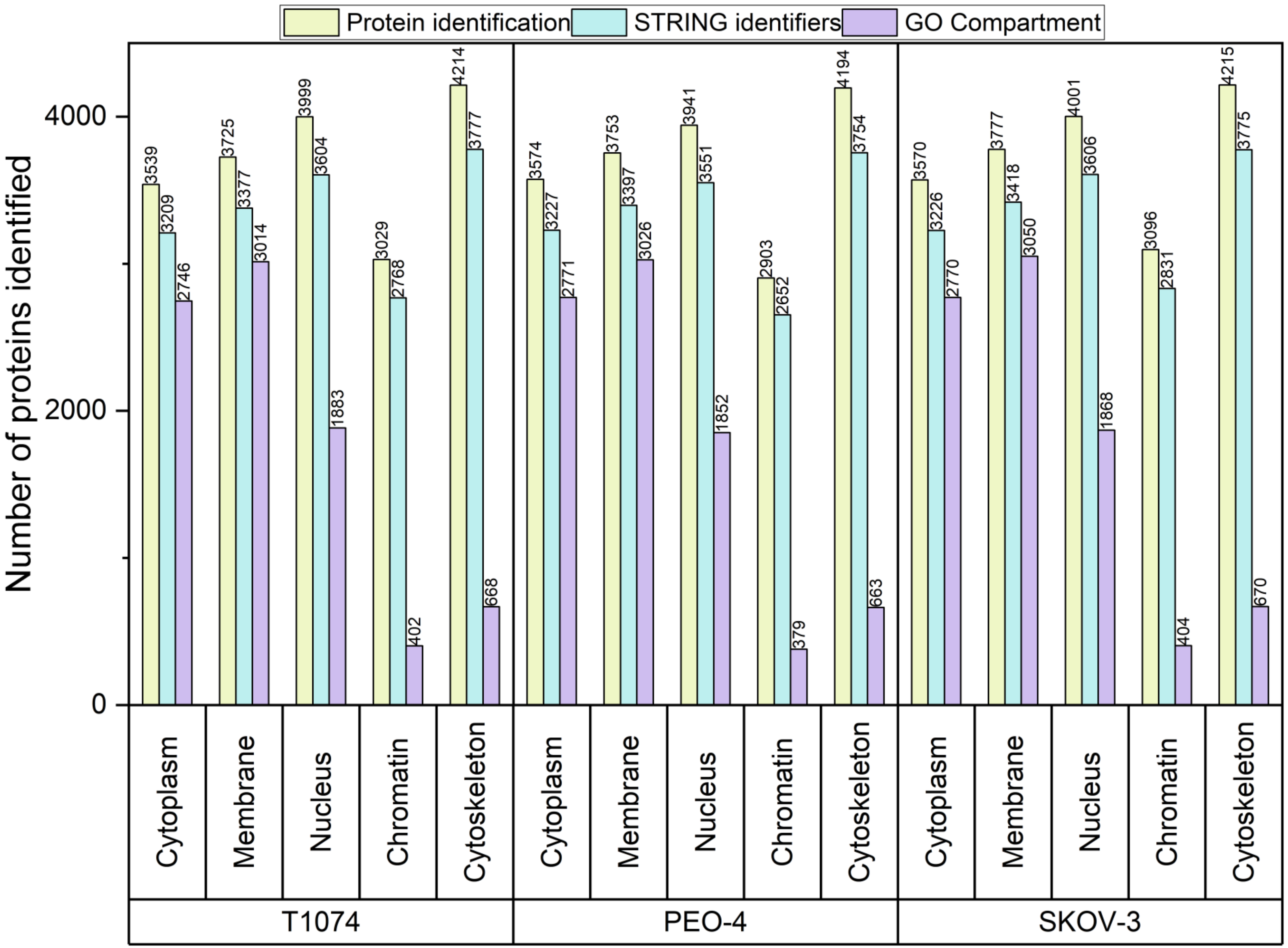

Supplemental Figure 6. (A) Hierarchical clustering heatmap showing the different RefProt clusters that can be observed among the three cell lines. Z-score range from -1.349 (green) to 1.307 (red).(B) Hierarchical clustering heatmap showing the different novel isoforms clusters that can be observed among the three cell lines. Z-score range from -1.348 (green) to 1.273 (red).

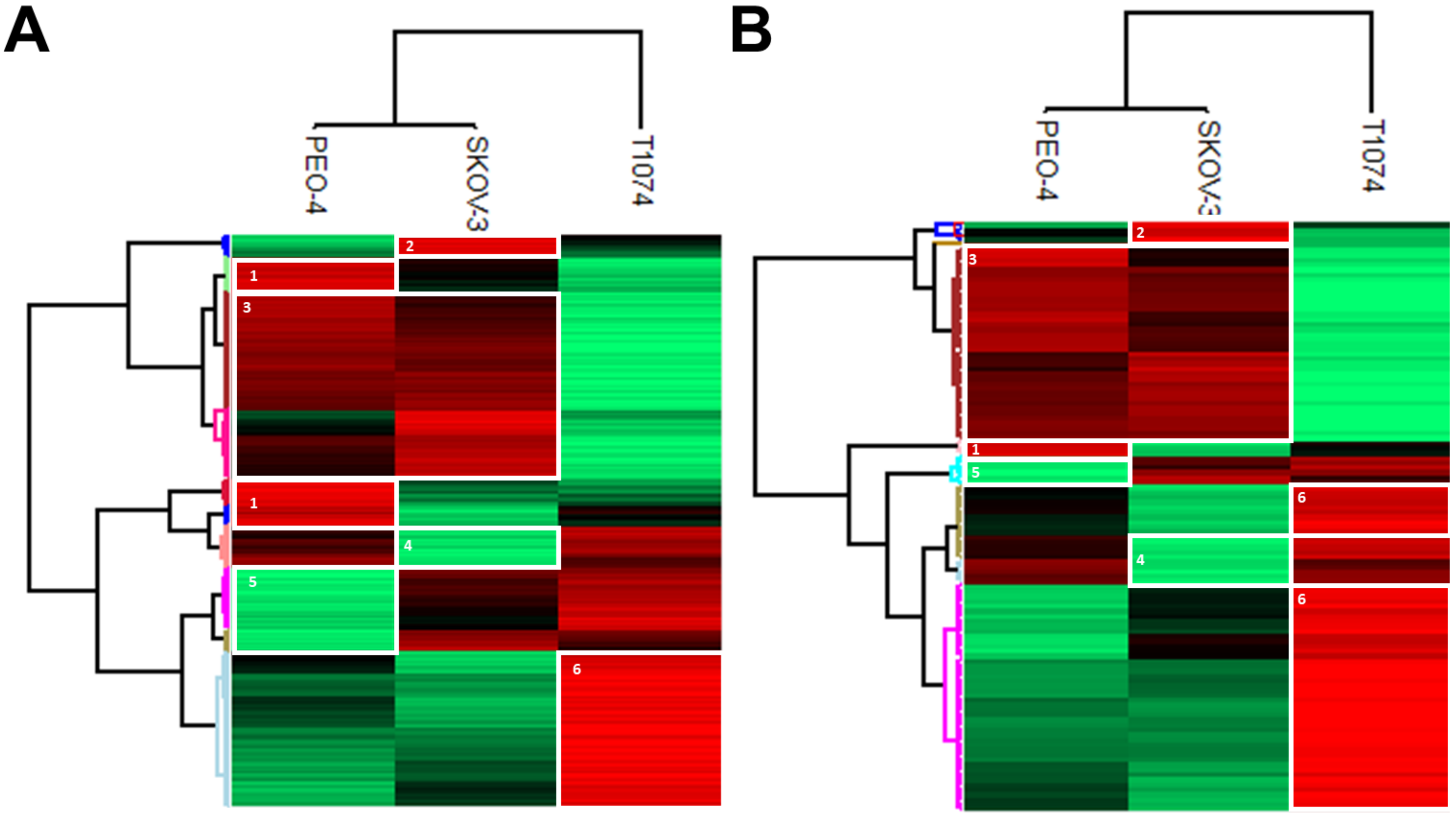

Supplemental Figure 7. Raw cross-link network in which AltProts are marked in orange and RefProts are marked in blue. Cross-links are marked in dark blue (SKOV-3 cells), purple (PEO-4 cells) and green (T1074 cells) dashed lines.-

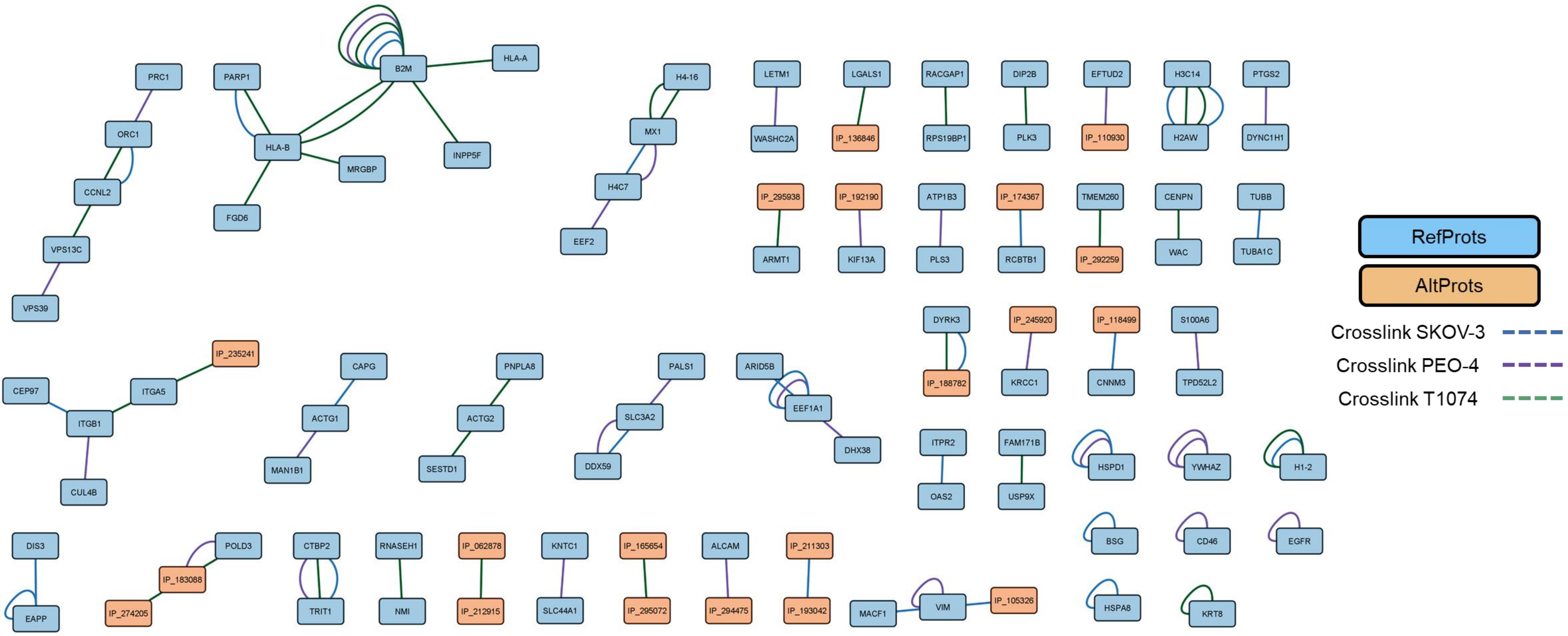

Supplemental Figure 8. Cross-link network in which AltProts are marked in orange and RefProts are in blue. Cross-links are marked in dark blue (SKOV-3 cells), purple (PEO-4 cells) and green (T1074 cells) dashed lines. Enriched by the STRING interactions (gray lines) retrieved. For the RefProts that did not present a referenced STRING interaction, an enrichment was performed to expand the network.

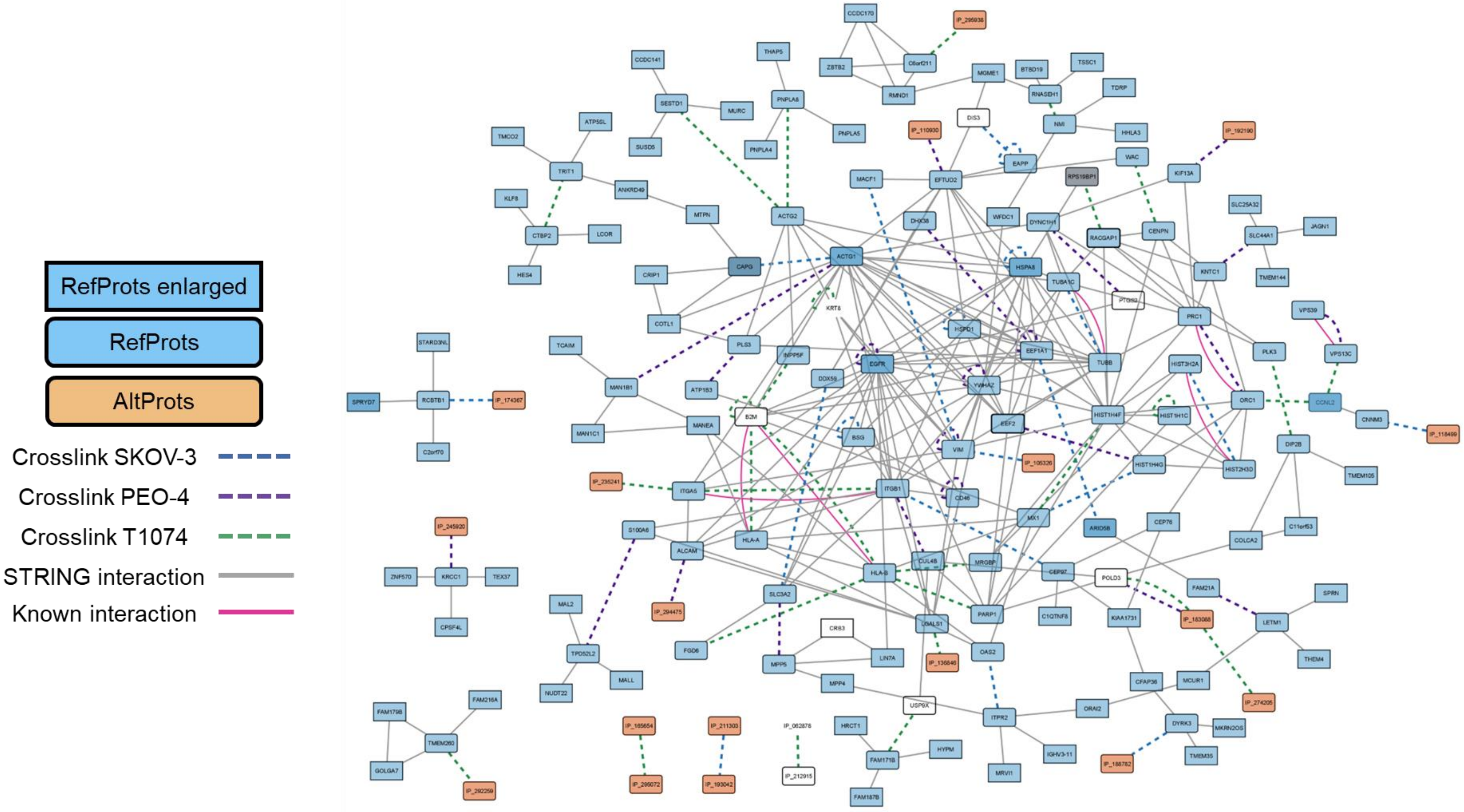

Supplemental Figure 9. Predicted interaction models docked in ClusPro for the RefProts (blue) and AltProts (orange). The distance between the residues Cross-linked are given for each interaction.

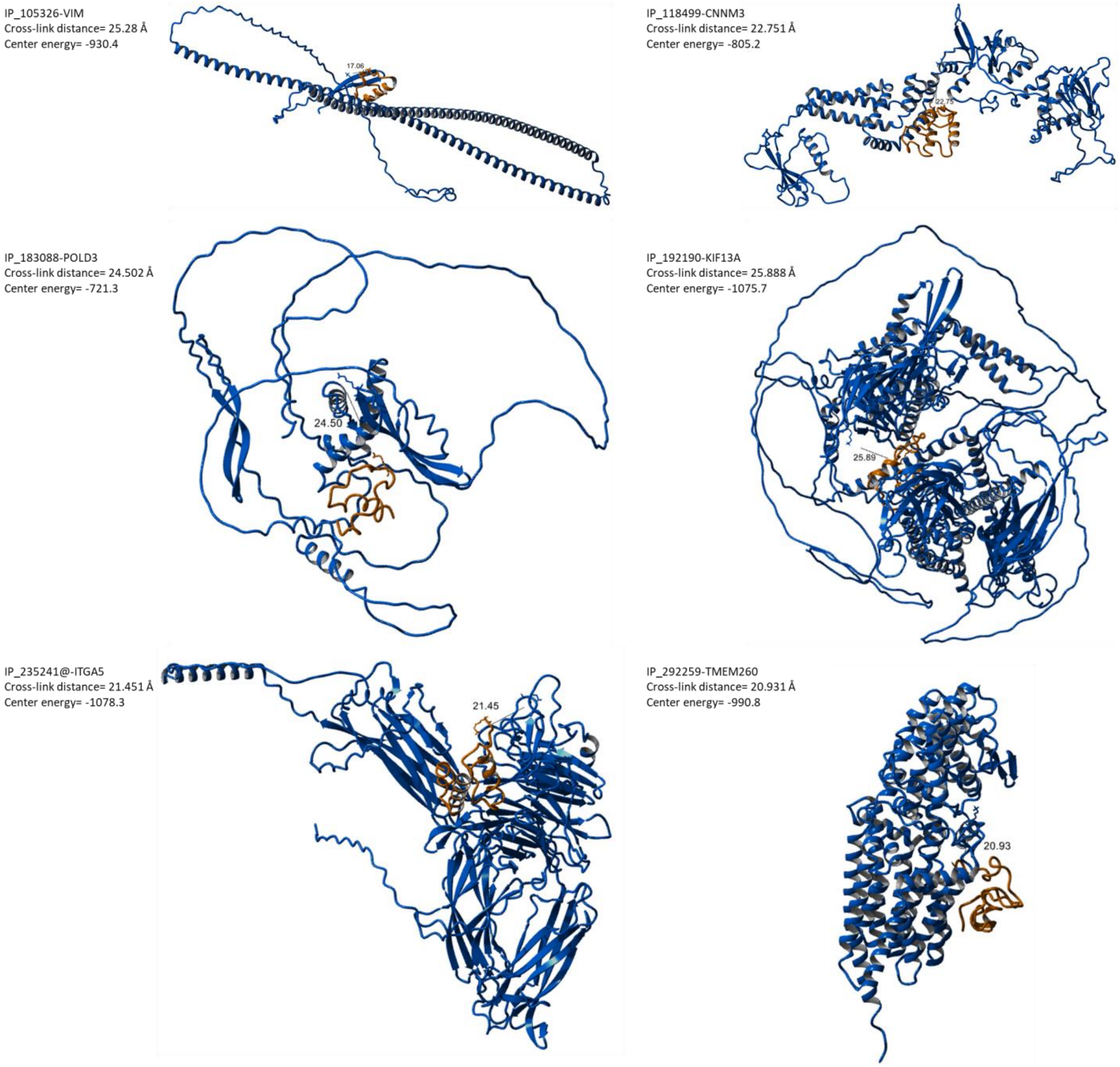

Supplement: Supplementary file 1 — Supplemental Figures [file 41419_2024_7046_MOESM1_ESM.pdf]
